# Leptin and psychiatric illnesses: does leptin play a role in antipsychotic-induced weight gain?

**DOI:** 10.1186/s12944-020-01203-z

**Published:** 2020-02-07

**Authors:** Francky Teddy Endomba, Aurel T. Tankeu, Jan René Nkeck, Joel Noutakdie Tochie

**Affiliations:** 1 Psychiatry Internship Program, University of Bourgogne, 21000 Dijon, France; 2 Department of Internal Medicine and sub-Specialties, Faculty of Medicine and Biomedical Sciences, University of Yaoundé I, Yaoundé, Cameroon; 3 Aging and Metabolism Laboratory, Department of physiology, University of Lausanne, Lausanne, Switzerland; 4 Department of Anaesthesiology and Critical Care Medicine, Faculty of Medicine and Biomedical Sciences, University of Yaoundé I, Yaoundé, Cameroon; 5 Human Research Education and Networking, Yaoundé, Cameroon

**Keywords:** Mental health disorders, Antipsychotic drugs, Second-generation antipsychotics, Antipsychotic-induced weight gain, Leptin

## Abstract

Antipsychotic-induced weight gain is the most prevalent somatic adverse event occurring in patients treated by antipsychotics, especially atypical antipsychotics. It is of particular interest because of its repercussion on cardiovascular morbidity and mortality especially now that the use of second-generation antipsychotics has been extended to other mental health illnesses such as bipolar disorders and major depressive disorder. The mechanism underlying antipsychotics-induced weight gain is still poorly understood despite a significant amount of work on the topic. Recently, there has been an on-going debate of tremendous research interest on the relationship between antipsychotic-induced weight gain and body weight regulatory hormones such as leptin. Given that, researchers have brought to light the question of leptin’s role in antipsychotic-induced weight gain. Here we summarize and discuss the existing evidence on the link between leptin and weight gain related to antipsychotic drugs, especially atypical antipsychotics.

## Introduction

Due to their various neurobiological actions, second-generation antipsychotics (SGAs) also known as atypical antipsychotics are increasingly used for the management of other mental disorders such as schizophrenia and related psychotic disorders [1, 2]. This growing use can be explained by the fact that the burden of mental health illnesses has greatly raised these past decades, placing psychiatric disorders as one of the main causes of morbidity and disability worldwide [2, 3]. According to the Global Burden of Disease Study (GBD) Collaborative Network, mental and addictive disorders affected more than 1 billion people globally in 2016 (approximately 16% of the world’s population), caused 7% of overall burden of disease as assessed in Disability-adjusted life years (*DALYs*) and 19% of all years lived with disability [2, 3]. Five types of mental illness appear in the top 20 aetiologies of GBD and for at least three of them, atypical antipsychotics can be indicated to control disease evolution and prevent relapse episodes [2, 4]. This includes schizophrenia, depression especially treatment-resistant depression or depression with psychotic symptoms, and bipolar disorders [2, 4]. Nonetheless, many concerns have been raised on their safety and tolerability especially regarding cardiovascular and metabolic health [4, 5]. A large number of scientific works have assessed the impact of second-generation antipsychotics on cardiometabolic features with major findings being an increase in morbidity due to QT prolongation with greater risk of sudden cardiac death, increased risk of stroke and coronary heart disease, insulin resistance with a higher risk of developing diabetes [4–6]. Antipsychotic-induced weight gain (AIWG), frequently encountered in patients treated with SGAs could be the common denominator despite theories demonstrating direct mechanisms of SGAs on the occurrence of stroke, coronary heart disease and type 2 diabetes [5, 7].

SGAs increased the risk of AIWG compared to the first generation and other psychotropic drugs. The degree of weight gained from SGAs is often greatest early in treatment and a meta-analysis showed that mean weight increases from 1.4 to 11 lb. (0.6–5 kg) over the initial 4–12 weeks of therapy [7, 8].There are numerous and interconnected underlying mechanisms [7, 8] comprising increased appetite, reduced basal metabolism and physical inactivity (probably linked to the sedative effect of antipsychotic drugs), associated with patients specificities such as gender or genetic variants [7, 9, 10]. Regarding the increase in food intake, it has been shown that atypical antipsychotics can modulate metabolic homeostasis in the hypothalamus, through effects on receptors of neurotransmitters such as serotonin (5-hydroxytryptamine 2c, 1b, 1a, 6), dopamine (D2), histamine (H1 and H3), adrenaline (alpha 2) and acetylcholine (M3) [7, 11–13]. Nevertheless, the exact mechanism of weight gain appears to be a more complex association of various neurobiological and metabolic pathways [14]. However, aside from these widespread and well-known theories on the pathophysiology of AIWG, there is growing interest in the implication of hormones involved in the food intake process, such as leptin [14, 15]. Identified 25 years ago, leptin (from the Greek *leptos*, meaning thin), greatly changed the perception of the adipocytes as storage cells [16, 17]. This 16-kDa peptide hormone predominantly secreted by adipocytes can reduce or suppress food intake and thereby induces weight loss [16, 17]. Hence we generated this mini-review which aimed to provide concise existing data and evidence on leptin metabolisms implication in the pathophysiology of AIWG.

## Basics on antipsychotics and leptin

### Antipsychotics and antipsychotics-induced weight gain

Described for the first time in early the 1950s, with chlorpromazine’s synthesis by the French Chemist Paul Charpentier and its presentation to psychiatrists by the surgeon Henri Laborit, antipsychotic properties were initially dedicated for the treatment of schizophrenia and related psychotic disorders [18, 19]. The discovery of chlorpromazine (at that time labeled as a tranquilizer) was followed nearly 10 years later by the introduction of other first-generation antipsychotics (FGAs), which helped to control some severe mental illnesses, especially those with “positive” symptoms [19]. Indeed FGAs were firstly designed for a sedative role but also an anti-delusional effect [18, 19]. However, the deleterious impact of FGAs on the cognitive, affective and motor domain, established the need to develop other pharmacological options [4]. Thereby in the 1980s, clozapine was developed and later in the 1990s and 2000s, there was the setting up of other drugs, differently acting from FGAs and currently named SGAs or atypical antipsychotics [18, 19]. At the time, neuroleptics (nowadays called antipsychotics) were mostly classified according to their clinical effects or their chemical structure [20, 21]. For instance, Deniker and Ginestet in 1971 proposed four categories of neuroleptics classification according to the clinical effects, including disinihibiting action and sedation [20]. Thus they distinguished sedatives neuroleptics (levomepromazine and chlorpromazine), average neuroleptics with both moderate therapeutic and moderate adverse effects (thioridazine and propericiazine), anti-delusional neuroleptics (sulpiride, prochlorperazine), and polyvalent neuroleptics with both sedative and disinihibiting properties (haloperidol, pipotiazine, fluphénazine) [20, 21]. However, actually, antipsychotics are much more classified based on their mechanisms of action, with the implication of various brain neurotransmitters and their receptors.

**Fig. 1:**
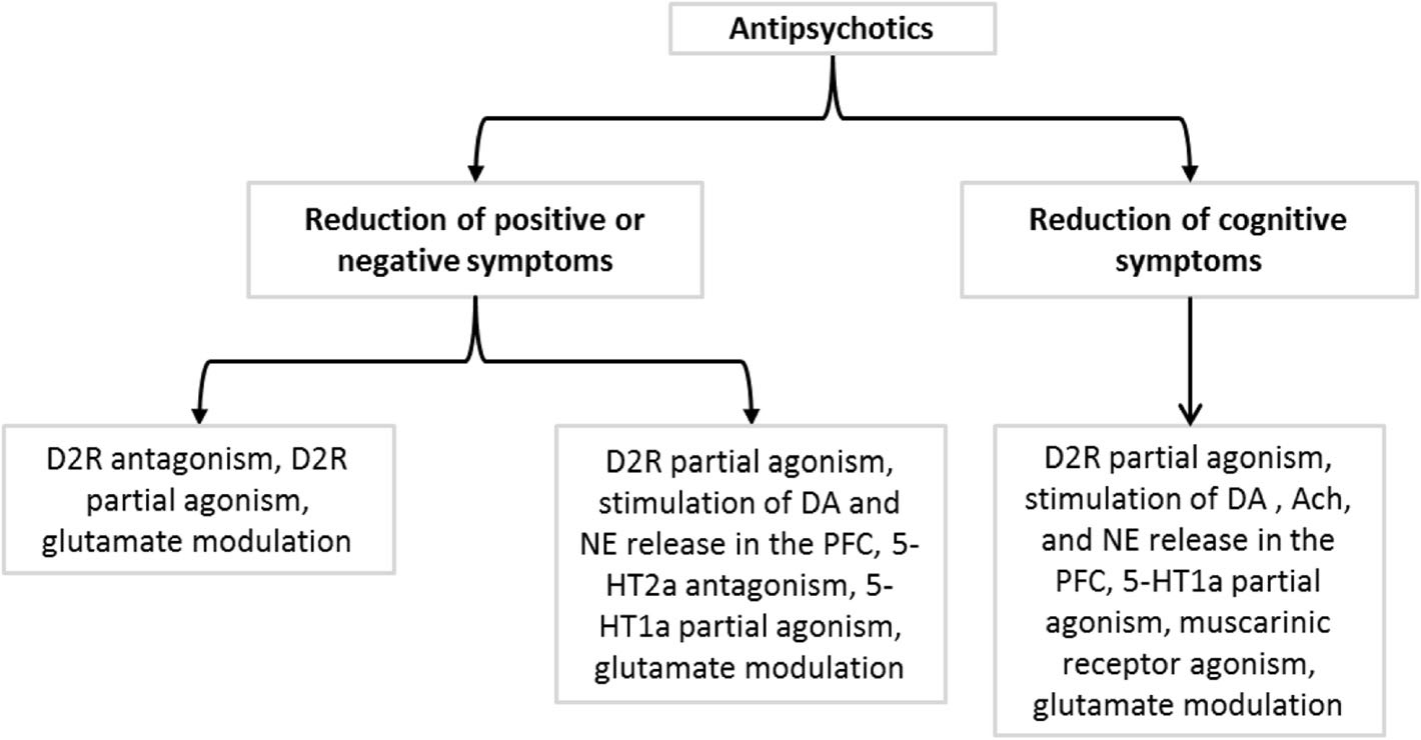
Neurobiological actions of antipsychotics and therapeutic effects. Ach: acetylcholine, DA: dopamine, HT: Hydroxytryptamine, NE: norepinephrine, PFC: prefrontal cortex

All first-generation antipsychotic agents have the common effect to produce high blockade of dopamine receptors (D2), due to their high affinity for this receptors type [21, 22]. Noteworthy, benzamides (with sulpiride as a leading molecule), which are sometimes considered as the first atypical antipsychotics (AAPs), have also action on dopamine D3 receptors [22, 23]. Atypical antipsychotics, as far as they are concerned, exhibit an action on other sites than dopamine D2 receptors. These sites include other dopamine receptors (D1, D2, D4), serotonin receptors (5-HT1a, 5-HT1d, 5-HT2c, 5-HT6, 5HT7), muscarinic cholinergic receptors (M3), histamine receptors (H1) and adrenergic receptors (alpha1 & alpha2) [22–24]. SGAs predominantly act by D2R antagonism, D2R partial agonist, dopamine, acetylcholine, and norepinephrine release in the prefrontal cortex (PFC), 5-HT2a antagonism, 5-HT1a partial agonist, muscarinic receptors antagonist or agonist, and glutamate modulation (Fig. 1) [23, 24].

For this last, it has been proved that some of the SGAs (for example clozapine and olanzapine) can selectively antagonize the effects of experimentally induced NMDA (*N*-methyl-D-aspartate) receptor hypofunction at behavioural levels [25]. The “atypicality” of SGAs, as hypothesized by Meltzer et al. in late the 1980s, is mainly defined by receptor occupancy [26]. Indeed, SGAs, inversely to most of FGAs, display the common singularity to occupy 5-HT2 receptors much more than they do with D2 receptors [22–24].

One other particular point to note is that aripiprazole, an atypical antipsychotic drug, differs in that it achieves diminished D2 receptor stimulation through partial agonist, thus reducing presynaptic DA release, and diminished activation of postsynaptic D2 receptors because of its weak intrinsic agonist activity [23, 24].

SGAs and FGAs differ also in the occurrence of adverse effects. While FGAs lead more frequently to extrapyramidal syndrome (EPS) and tardive dyskinesia (TD) compared to SGAs, SGAs are more associated with metabolic disturbances than FGAs [4, 6, 22–24]. Indeed for some authors, SGAs can be most accurately and simply described as one that produces minimal extrapyramidal syndrome at clinically effective doses [22–24]. Extrapyramidal syndrome and tardive dyskinesia, as well as a neuroleptic malignant syndrome, has been proved to be linked to blockade of dopamine D2 receptors in the dorsal striatum [23, 24]. One other metabolic effect, also related to D2 antagonism and unspecific to first or second generation APD is elevated plasma prolactin [22–24].

As previously mentioned, APD, especially atypical ones, are associated with a substantial risk of metabolic abnormalities, including disturbances of lipid metabolism, obesity, and diabetes mellitus [4, 6, 23]. These metabolic side effects are primarily the result of altered energy (food) intake, which mechanisms remain complex [4–6, 24]. Weight gain induced by SGAs can be responsible for suboptimal medication compliance and high rates of discontinuation, thus leading to the possibility of symptomatic relapse and poor long-term outcomes [7, 8, 23, 24]. Also, AIWG, through cardiovascular risk upswing, can result in reduced life expectancy of patients due to acute complications such as myocardial infarction and stroke, and chronic conditions including heart and kidney failure [4–6]. Noteworthy, among atypical antipsychotics, clozapine and olanzapine have been proved to more frequently induce metabolic disturbances and weight gain, while demonstrating the highest clinical efficacy regarding psychiatric symptoms [7, 24].

Numerous previous studies demonstrated AIWG results from a combination of excess caloric intake, associated with increased water weight, but despite years of study, the molecular mechanisms remain unclear [4–6, 11–13]. Nevertheless, studies done on the topic, especially on rodents’ model, demonstrated increased expression of orexigenic peptides (neuropeptide Y and agouti-related protein) and decreased expression of anorexigenic one (proopiomelanocortin) in response to some SGAs, notably olanzapine [7, 8, 11]. The effect of AAPs on body weight has been proved to be sustained by the action on various receptors. This includes the inhibitory effect on melanocortin receptor 4 (MC4R4), as well as on histamine receptor 1 (H1R), some serotonin receptors (5-HT2c and 5-HT1b) and dopamine receptor 2 (D2), and the stimulatory effect on muscarinic receptors (M3) as well as on some serotonin receptors (5-HT1a and 5-HT6) and adrenergic ones (α2) [11–14]. Apart from direct action on food intake through various brain neurotransmitters, several other hypotheses have been aroused to explain metabolic disturbances, particularly weight gain, induced by SGAs [10, 27, 28]. We so have several observations showing decreased energy expenditure linked to the sedative effect of SGAs [10, 27]. Indeed, for this last, a recent systematic review investigating the role of the gut microbiome on metabolic alterations pertaining to SGAs concluded that AAPs’ related microbiome alterations potentially result in body weight gain [28]. However, also aiming to understand how APD and more specifically SGAs induce weight gain, researchers explored the field of food intake regulatory hormones such as leptin.

### Leptin

Leptin is a peptide produced by adipocytes proportionally to fat stores that controls food intake and energy homeostasis through central mechanisms, mediated by a network of neuropeptides at the hypothalamic level [17, 29]. It acts as a hormone and a cytokine, and can also be secreted in lower amounts by the mammary gland, ovary, skeletal muscle, stomach, pituitary gland and lymphoid tissue [29, 30]. Interestingly, the hormonal and metabolic effects of leptin were discovered several years before it’s the discovery of leptin [30]. Indeed, in 1950 was described the first leptin-deficient mouse (*ob−/ob−*), a mutant strain characterized by morbid obesity and decreased rates of basal metabolism [30, 31]. The gene coding for this peptide is known as the human obese gene (*OB*), and located on chromosome 7 (7q31.3). Its actions are mediated by the leptin receptor (LepR), mainly expressed in the human brain (especially the hypothalamus and cerebellum) whose gene is located on chromosome 1 [29–31]. The adipocyte-secreted leptin exhibits a circadian secretion rhythm, with higher secretion at night and lower ones during daytime [29–32]. Factors influencing the level of circulating leptin include energy stores, food intake, gender, age, physical activity and glucose uptake [32].

**Fig. 2:**
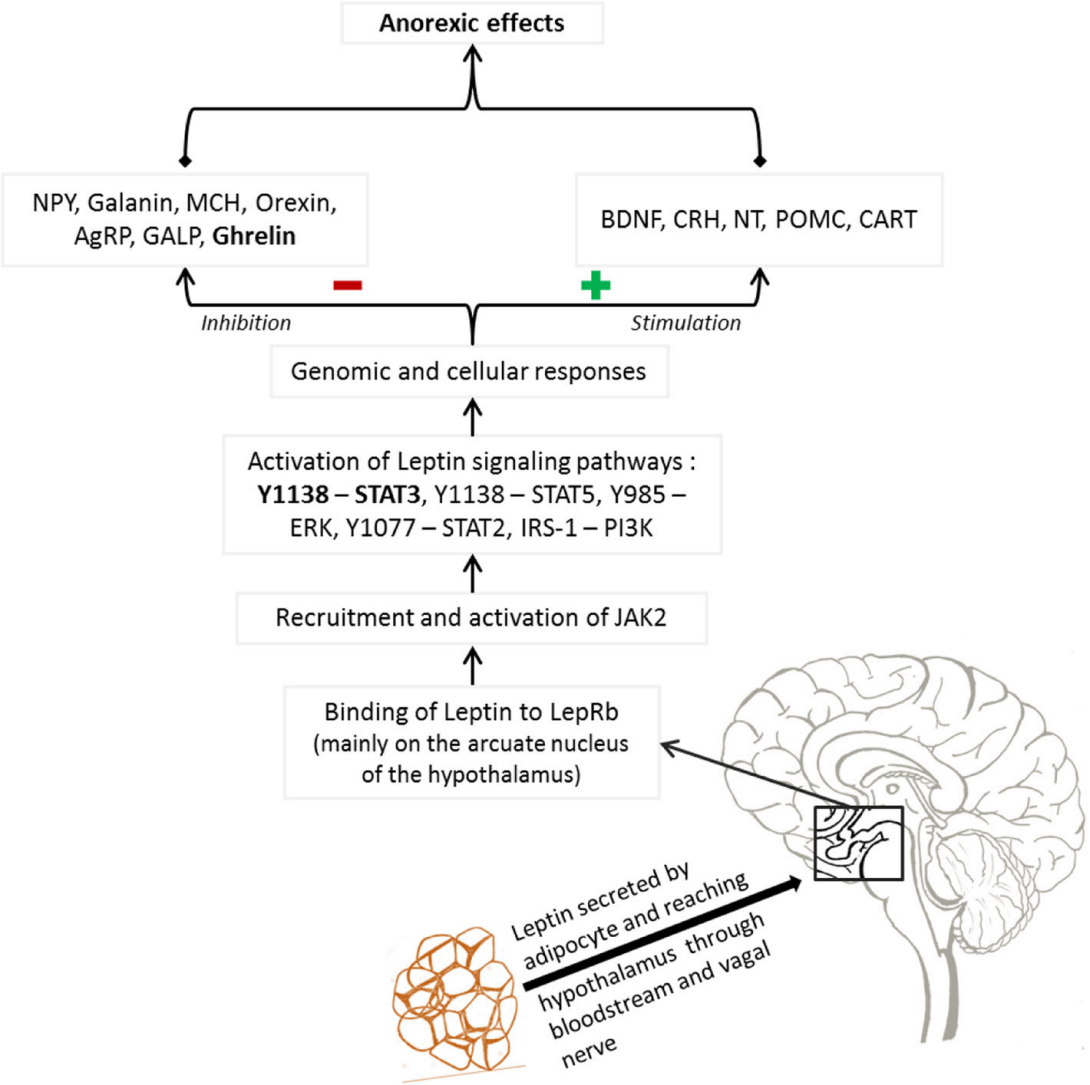
Pathways of energy balance control by leptin. AgRP: agouti-related protein, BDFN: brain-derived neurotrophic factor, CART: cocaine- and amphetamine-regulated transcript, CRH: corticotrophin-releasing hormone, ERK: extracellular signal-regulated kinases, JAK: Janus activated kinase, GALP: galanin-like peptide, IRS-1: insulin receptor substrates, LepRb: leptin receptor isoform b, MCH: melanin-concentrating hormone, NPY: neuropeptide Y, NT: neurotensin, PI3K: phosphatidylinositol 3-kinase, POMC: pro-opiomelanocortin, STAT: signal transducer and activator of transcription

Once produced, leptin reaches the brain by the bloodstream and vagal nerve, and enter it by transcytosis across the blood-brain barrier (BBB), through the mechanism of saturating transport [33]. This is because leptin’s size is too great to cross by diffusion, and therefore leptin requires and adjustable and saturable transport system [33, 34]. After crossing the BBB, leptin fixes its receptors located in the hypothalamus, particularly the arcuate nucleus (ARH) [33]. Leptin receptors belong to the class 1 family of cytokine, and currently, six isoforms of LepR are known: LepRa, LepRb, LepRc, LepRd, LepRe and LepRf [33–35]. All these receptors are known to have a common leptin-binding domain but to differ in their intracellular domains [33–35]. Only the long isoform (LepRb) can display leptin-mediated cell action through several signaling pathways [34, 35], the role of the other isoforms being not well understood. As illustrated in Fig. 2, the binding of leptin to LepRb leads to the recruitment and the activation of JAK2 (Janus Activated Kinase 2), which allows autophosphorylation associated with phosphorylation of many intracellular residues, notably Y985-ERK, Y1077-STAT2, Y1138-STAT3, STAT5 and IRS-1 (Insulin Receptor Substrates) [35, 36]. The phosphorylated residues therefore trigger the recruitment of signaling pathways as follow: Y1138 for the STAT3 (Signal Transducer and Activator of Transcription) and STAT5 pathways, Y985 for the ERK (Extracellular signal-regulated Kinases) pathway, Y1077 for the STAT2 pathway, and IRS-1 for the PI3K (Phosphatidylinositol 3-Kinase) pathway [34–37]. All these signaling pathways contribute to leptin-mediated cell actions, mostly dominated by food intake (anorexic effects) and energy homeostasis [36, 37]. Leptin also stimulates the activation of the regulatory protein SOCS3 (suppressor of cytokine signaling 3) and PTP1b (protein tyrosine phosphatase 1B), and both inhibit leptin signaling [32, 38]. Indeed, SOCS3 are negative regulators of the JAK/STAT pathway and initiate a negative feedback loop with the inhibition of further phosphorylation/activation of JAK2 [32, 38]. However, on the precise mechanism by which protein tyrosine phosphatase 1B regulates leptin signaling, there are still ongoing research works [32, 38].

Leptin acts on energy homeostasis by modulating production/action of anorexigenic peptides such as pro-opiomelanocortin (POMC), cocaine- and amphetamine-regulated transcript (CART), neurotensin (NT), corticotrophin-releasing hormone (CRH) and brain-derived neurotrophic factor (BDNF) [17, 32] (Fig. 2). More precisely, leptin affects food intake by acting on two types of neuronal populations [32, 38]. Firstly, we have neurons activated by leptin, that co-express POMC and CART (ARH POMC/CART neurons, predominantly located in the lateral part of ARH), and suppress food intake [32, 36, 37]. POMC can be cleaved and formed numerous peptides including α-melanocyte-stimulating hormone (α-MSH), which can stimulate melanocortin receptors 3 and 4 (MC3R and MC4R) [32]. Previous studies demonstrated that structural alterations of the MC4R receptor and the insufficient number of MC3R receptors in mice models can result in leptin resistance and obesity [32, 38].

On the other hand, we have neurons predominantly located in the ventromedial part of ARH, inhibited by leptin, that co-express neuropeptide Y, agouti-related peptide (AgRP) and gamma-amino butyric acid (GABA), and induce food intake (Fig. 2) [32, 39]. Overall, leptin action on energy metabolism leads to decreased appetite, weight loss and changes in endocrine function and metabolism [17, 32, 38].

According to all that, and as previously demonstrated in the literature, leptin deficiency can easily lead to obesity, but obesity-related to leptin metabolism can also be the result of deleterious leptin’s action [32, 37]. Indeed, even with increased leptin levels in the blood, the efficacy or more particularly the anorexic effect of leptin can be diminished in the case of leptin resistance [35, 37]. Although clear criteria for the definition of leptin resistance and diagnosis have not yet been established, several potential underlying mechanisms have been identified [32, 35]. We so have altered leptin transport across the blood-brain barrier, dysregulation of leptin expression, hypothalamic inflammation, endoplasmic reticulum stress, autophagy disorders, peripheral inflammation (given the functional and anatomical relationship between adipocyte and lymphoid cells and also the proinflammatory properties of leptin) and gene mutation (especially the OB gene) [32, 35].

**Fig. 3:**
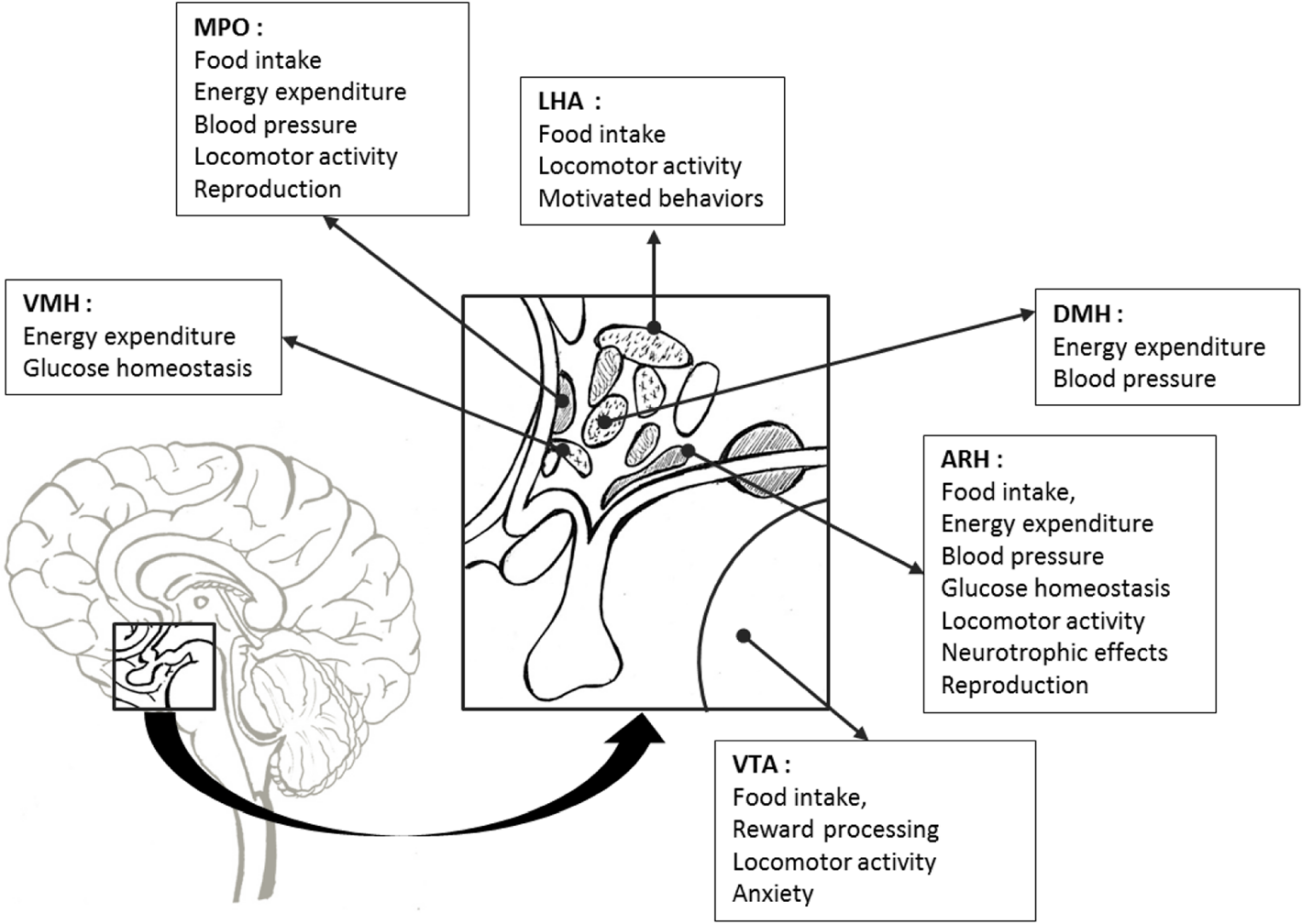
Areas of LepR-expressing neurons and leptin-mediated biological functions. ARH: arcuate nucleus of the hypothalamus, DMH: dorsomedial nucleus of the hypothalamus, LHA: lateral hypothalamic area, MPO: medial preoptic area, VMH: ventromedial nucleus of the hypothalamus, VTA: ventral tegmental area

Apart from the ARH, other regions that contain LepR-expressing neurons, and thus can lead to leptin biological actions through the transmission of leptin signaling (Fig. 3) [40].

Initially famous for its key role as a hormone, regulating body mass homeostasis, with its suppressive effect on food intake and its boosting action on metabolic rate, more recently, leptin’s metabolism has been involved in many other processes and diseases [41, 42]. This encompasses cancers, due to its pro-carcinogenic effects with action on cell development and suppression of apoptosis, neurodegenerative diseases, by its neuroprotective and neurotrophic effects in Alzheimer’s disease, and some mental illnesses, including psychotic and mood disorders [41, 42].

## Leptin and psychiatric illnesses

Modifications of leptin metabolism and its gene expression, as well as its receptor, have been reported among patients with mental health disorders, independently to psychotropic drugs or in absence of antipsychotics [43]. Several studies showed a particular interest in the relationship between leptin and depressive disorders, but with discordant conclusions [42–45].

To begin, authors claiming that depression are associated with low leptin levels, thus suggesting a positive association/correlation between leptin levels and improvement in the depressive mood [43, 45]. Indeed, pharmacological studies showed that intra-hippocampus administration of leptin can exert an antidepressant-like effect, thus demonstrating that leptin regulates dopaminergic neurotransmission in mesolimbic areas [45, 46]. These studies on animal models found that leptin reduces symptoms of depression and had an anxiolytic effect through modulation of the hypothalamic-pituitary-adrenal axis (HPA) [45, 47]. This axis is part of the networks that regulate mood, emotional behaviour as well as other specific functions like rewards processing [47, 48]. These networks, studied by functional neuroimaging, neurobiology, and neuropathology, also include the medial prefrontal cortex, the caudolateral orbital cortex, the amygdala, the hippocampus and ventromedial parts of the basal ganglia [46]. All these areas have also been proven to have an important role in feeding behaviour and nutrition hormones metabolism [30]. Also, as previously mentioned, leptin has stimulating function on brain-derived neurotrophic factor activity (BDNF) [32, 45, 49]. As support by current evidence, BDNF plays a key role in the pathophysiology of major depression through neuroplasticity and neurogenesis and is at the base of the classic neurotrophic hypothesis of depression [50, 51]. Neural plasticity refers to the ability of a neuron or the brain to adapt to external or internal stimuli, involving a series of cellular events, including neurogenesis, cell migration, cell survival, synaptogenesis, and the modification of mature synapses [52]. Neural plasticity is also one of the therapeutic targets of novel and promising depression treatments, such as esketamine [53]. Esketamine is a non-competitive antagonist of glutamate receptors *N*-methyl-D-aspartate (NMDA), and leptin has been found to act on this receptor by directing its synaptic activity [54]. Concerning neurogenesis, previous animal (mice) studies demonstrated that leptin plays a critical role in neuroprotection especially by the regulation of the GSK-3β/β-catenin signaling pathway [55, 56].

On the other hand, some studies found that patients with depressed mood had higher levels of leptin than a control group [45]. A recently published study analysing plasma levels of leptin in young adults revealed a positive association between plasma leptin levels and self-reported depressive symptoms, especially for women [57]. Indeed, several previous studies displayed a clear sex difference of leptin levels, with females having higher amount than males [57–59]. Carvalho et al. in their meta-analytic study published in 2014, found higher peripheral leptin levels in participants with mild to moderate major depressive disorder compared with controls, but no significant difference between controls and severely depressed patients [60]. However, a recent meta-analysis concluded that there is no difference in leptin levels between depressive subjects and controls [61]. Confounders such as age, gender-associated metabolic disturbances, medication history and clinical type of depressive disorders, might impact peripheral leptin levels, and thereby justify these inconsistent results [42, 45]. Some other studies showed that leptin levels are increased in major depressive disorders, but only in patients experiencing atypical features, suggesting that leptin may be involved in a subset of patients with increased weight [62, 63]. Concerning bipolar disorder, a meta-analysis conducted in 2016, including eleven studies and 1118 participants, provides evidence that leptin levels are not altered in BD when compared to healthy controls [64].

Studies on the relationship between, psychotic disorders, especially schizophrenia, also present divergent results [65, 66]. Indeed, two recent meta-analyses showed an increase in leptin for schizophrenia patients compared to controls, with more marked elevation during decompensations episodes [67, 68]. Martorell and colleagues, in their study published in 2019, detected increased leptin levels in the early stages of psychosis [69]. They compared 39 first-episode psychosis (FEP) patients, 32 psychotic patients in the critical period and 21 healthy controls [69]. They also found significant correlations between leptin levels and anthropometric, lipid, hormone, and cytokine parameters [69]. In a meta-analysis including 1674 patients and 2033 controls, Stubbs and collaborators reported increased leptin levels in patients with mean illness duration of 9.3 years [67]. On the other hand, a recent meta-analysis revealed that impaired appetite regulation, in terms of elevated insulin levels and decreased leptin levels, occurs in early psychosis, before antipsychotic treatment [70]. The raised leptin levels found in some studies among schizophrenia patients could be explained by the negative feedback against increased in brain dopamine activity associated with positive symptoms since leptin has been proved to modulate mesolimbic dopamine system [45, 67]. This modulation takes into account the inhibitory action dopamine neurons in the ventral tegmental area and the promotion of tyrosine hydroxylase expression [45, 67].

## Leptin and antipsychotics-induced weight gain

These last three decades, a significant number of studies have paid particular attention to serum leptin levels modifications with antipsychotic drugs, especially atypical antipsychotics with the main studied SGAs being clozapine, olanzapine, risperidone and quetiapine [14, 71, 72]. Nonetheless, we still actually don’t know if antipsychotics medications induce leptin elevation by a direct mechanism or through weight gain.

According to published literature, several studies tend to attribute serum leptin elevations in patients with SGAs medication, to weight gain rather than the direct effect of these drugs on leptin metabolism [14, 73]. A meta-analysis performed in 2015 including 39 studies that reported levels of leptin before and after antipsychotic regimen in patients followed for schizophrenic disorders, revealed a positive and moderate effect size, with a significant positive association between leptin and body mass index (BMI) [72]. According to this analysis, olanzapine, clozapine, and quetiapine produced moderate leptin elevations; meanwhile, haloperidol and risperidone were associated with small and non-significant leptin changes [72]. Since leptin is released by the adipocyte and is therefore proportional to fat mass and stores, its increase in blood of patients with antipsychotic-induced weight gain could be the result of the increased weight itself suggesting that hyperleptinemic state could be more a consequence than a cause of antipsychotics-induced weight gain (AIWG) [14, 72–74]. Greater leptin elevation with olanzapine, clozapine, and quetiapine can be explained by the higher affinity of these drugs for muscarinic M3 receptors, which are known to be the underlying source of large differences concerning weight gain and other side effects [72]. Indeed, SGA-induced weight gain could be due to the stimulation of muscarinic M3 receptors as well as of 5-HT1a, 5-HT6, and adrenergic α2 receptors, and also the blockade of serotonin 5-HT2c and 5-HT1b, adrenergic α1, dopamine D2 and histamine H1 receptors [14, 75, 76]. The inhibitory effect on hypothalamic H1Rs includes activation of AMP-activated protein kinase (AMPK), a well-known feeding regulator [22–24, 76]. Noteworthy, suppressing effect on dopamine D_2_ receptors with and impact on reward processing and physical activity is also the mechanism by which FGAs can induce weight gain [4, 10]. According to prospective studies, an increase in serum leptin levels compared to baseline occurs a few hours after the first treatment administration (of SGAs), seems to peak between 6 and 10 weeks, and remains stable up to several months [77]. Another molecule that plays a role in antipsychotics-induced weight gain is 5-Hydroxytryptamine (5-HT) acting similarly to leptin, promoting the reduction in food intake and increasing energy expenditure [73, 78]. Also, 5-HT is classified as a brain satiety factor, and the action of SGAs at 5 HT-receptors, especially the suppressing effect on 5-HT2c receptors, could justify its implication in AIWG [79, 80]. Leptin has been shown to endorse central 5-HT turnover through nitric oxide (NO) dependent pathway, and it has been demonstrated that central leptin-induced anorexia is in part mediated by 5-HT2c receptor [4, 14, 73]. 5-HT2c receptors are alleged to influence metabolic function by regulating the transmission of melanocortin and its precursor POMC, which both display an anorexigenic action [19, 81]. Also, some effects of serotonin on leptin metabolism have been reported, with a dose-dependent increase in serum leptin observed in response to 5-HT [73, 78, 79]. Another possible interaction between antipsychotics action and leptin metabolism can be found through NPY activation, partially induced by the blockade of H1 receptors and reversed by H1 agonist [14, 82]. Indeed, olanzapine has been reported to enhance NPY expression, which is a triggering factor of adipocyte-leptin secretion [11–14, 82, 83]. Interestingly, in their study published in 2005, Haupt and colleagues argued against a role for defective leptin secretion, clearance, or signaling in the body weight gain induced by antipsychotics [84]. They compared leptin levels between 72 schizophrenia patients chronically treated with olanzapine (27), risperidone (24) or typical antipsychotics (21) and 124 healthy adult control subjects [84]. They found that adiposity-related elevations in plasma leptin concentrations in antipsychotic-treated patients with schizophrenia are highly comparable to those observed in untreated healthy control subjects [84]. At the same time, this study didn’t either measure peripheral leptin effects or collected prospective serial measurements of plasma leptin at the initial phase of the treatment, thus justifying the need for more precise research works to confirm their findings [84]. However, 5 years later Wilmsdorff et al. through a work assessing the impact of typical and atypical antipsychotic drugs on leptin concentration and changes in the receptor expression in the hypothalamus of male rats concluded that the drugs didn’t act directly on the leptin regulatory system [85]. The studied drugs were haloperidol, clozapine, and ziprasidone, and were compared to a control group [85].

On the other hand, numerous evidences suggested that antipsychotic drugs can have an action on leptin metabolism without going through the body weight gain pathway [81, 86–90]. For instance, Monteleone et al. found that in the first phase of clozapine treatment, there is a marked rise in circulating leptin independently of total BW changes [86]. They hypothesized that this elevation could theoretically signal to the brain the immediate need for appetite suppression and/or increase in the energy expenditure to counteract the incoming disturbance of the metabolic state induced by clozapine [86]. Recently, Tsubai et al assessed the expression of adipokines in mature 3 T3-L1 adipocytes cultured with clozapine and found a causal role for histamine H1/serotonin 5-HT2c receptors [91]. They concluded that clozapine, but not blonanserin, strongly and directly interacts with leptin secretion (as well as other adipokines’ secretion) and adipocytes enlargement. Thus, this drug’s effect on metabolic disturbances is also linked to a direct action on adipocytes mechanism of the regulation of food intake [91]. Indeed, in their study, short and long-term exposure to clozapine significantly decreased secretion of leptin and its mRNA expression [91]. More specifically, while investigating the mechanisms of this decreased metabolism of leptin pertaining to clozapine, the authors found that a selective serotonin 5-HT2c antagonism significantly enhances the lowered secretion of leptin triggered by clozapine when compared to controls [91]. According to their findings, this was not the case for histamine H1 antagonism [91]. Similarly, while studying the dynamics of serum leptin level and some anthropometric values in patients with schizophrenia and treated by risperidone, olanzapine and clozapine, Gorobets and collaborators’ results suggested that leptin resistance contributes to the pharmacogenetic increment of body weight [87, 92]. Piao and collaborators studied the effects of risperidone on leptin-stimulated STAT3 and found that this drug inhibits leptin signalling in the human SH-SY5Y neuroblastoma cell line through the induction of suppressor of signalling of cytokine 3 and 6 [93]. Here, it was shown that the administration of risperidone induces the enhancement of both SOCS3 and SOCS6 mRNA expression through ERK activation, and the inhibition of leptin-induced STAT3 phosphorylation [93]. They concluded in a possible mechanism of leptin resistance and consequently body weight gain induced by this AAP in patients followed for schizophrenic disorders [93]. Some studies also support the theory of an impact of leptin and leptin receptor genes polymorphism on AIWG, since mutations about these genes have been described to lead to obesity, hyperphagia and insulin resistance, as well as immune and reproductive disturbances [81, 89]. For instance, Brandl et al while investigating the influence of leptin and leptin receptor genes polymorphisms on weight modifications linked to SGAs in 181 schizophrenic or schizoaffective patients, found an impact of *LEP* (but not *LEPR*) gene variation on AIWG [89]. However, the authors proposed that large-scale studies with more homogeneous samples be conducted to enhance their results [89]. Indeed there are research works that didn’t found a significant association between AAPs, leptin, leptin receptor gene’s expression, and BMI or AIWG [94–96]. The polymorphism of the leptin gene promoter region is recognized to have an impact on leptin secretion, hence, to be associated with body weight gain and obesity [14, 97]. Yang et al. in a study involving 539 samples and which aimed to examine the -2548G/A functional polymorphism in the leptin gene promoter found that homozygosity for this polymorphism was significantly associated with AIWG [97]. Shen and colleagues realized for their part a meta-analysis study on the relationship between leptin -2548G/A gene polymorphism and AIWG and found discrepant correlations according to ethnicity [90]. Indeed in their study, which included 451 AIWG patients and 568 controls, there was a significant association between the -2548A allele and the risk of weight gain in Asian populations while in Europeans the -2548A allele seemed to decrease the risk [90]. It also has been hypothesized that AIWG may be due to desensitization of leptin receptors, as a possible consequence of genetic vulnerability involving 5-HT_2C_ receptor gene loci [88]. Another mechanism that can sustain the possibility of leptin resistance leading to body weight gain and AAPs is neuro-inflammation particularly in hypothalamic cell line. Indeed, hypothalamic inflammation is known to be one of the several mechanisms of leptin resistance [32] and a study realized by Kowalchuk et al. suggested an upregulation of proinflammatory pathways mediated by AAPs, especially olanzapine, clozapine and aripiprazole [98].

**Fig. 4:**
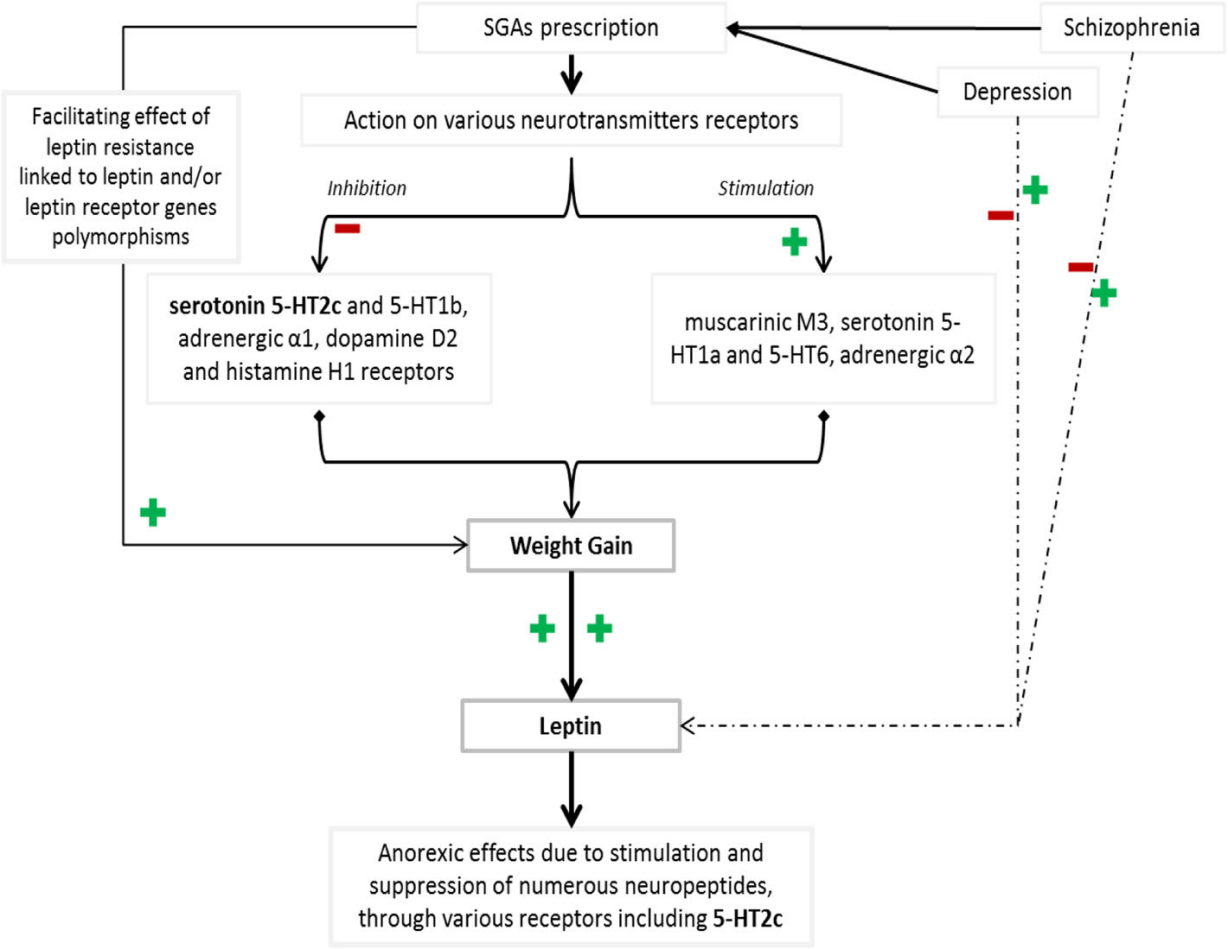
Interactions between SGAs, weight gain and leptin metabolism. D: dopamine, H: histamine, HT: Hydroxytryptamine, M: muscarinic, SGAs: second generation antipsychotics. The symbols (**+**) and (**−**) respectively refers to stimulation and inhibition

Despite the huge amount of evidence elucidated from good-quality studies, the correlation between leptin and leptin receptors metabolism and AIWG remains questionable. Considering the demonstrated fact that SGAs exhibit epigenetic effects [99, 100] and the association between leptin resistance and weight gain [32, 37], one possible mechanism of a direct link between AIWG and leptin could be epigenetic-induced changes on leptin or leptin receptor genes. In this case, epigenetic modifications induced by antipsychotics could induce or exacerbate a leptin-resistance status and thus generate metabolic conditions resulting in weight gain. Thereby, therapeutics targeting leptin metabolism would be of interest to patients with AIWG. These treatments would include for instance synthetic leptin receptor agonists (such as leptin 22–56 peptide), leptin sensitizing molecules (such as amylin), inhibitors of leptin signaling negative regulators (such as trodusquemine) and enhancers of leptin transport through the blood-brain barrier (such as pluronics) [101–103]. Figure 4 illustrates receptors which inhibition or stimulation leads to SGA-induced body weight gain, and also the current hypothesis that sustains interactions between antipsychotics induced weight-gain and serum leptin elevation.

## Conclusion

According to current existing literature, elevated serum leptin levels in psychiatric patients treated by antipsychotics, mainly SGAs, tend to be recognized as the consequence of the underlying mental disorders and/or antipsychotics induced weight gain, but not to be the cause of body weight gain. However, some recent findings sustain the possibility of another mechanism involving epigenetic-induced changes of antipsychotic drugs on leptin and/or leptin receptors genes. Future studies in the field might provide more information concerning the exact mechanism of raised leptin levels in patients with AIWG. This is of great interest since a possible implication of leptin in AIWG could be considered as a potential therapeutic target for the management of AIWG.

## Data Availability

Data sharing is not applicable to this article as no datasets were generated or analysed during the current study**.**

## Abbreviations

AAPs: Atypical antipsychotics
AgRP: Agouti-related peptide
AIWG: Antipsychotics-induced weight gain
ARH: Arcuate nucleus of the hypothalamus
BBB: Blood-brain barrier
BD: Bipolar disorder
BMI: Body mass index
BNDF: Brain-derived neurotrophic factor
CART: Cocaine- and amphetamine-regulated transcript
CRH: Corticotrophin-releasing hormone
EPS: Extrapyramidal syndrome
ERK: Extracellular signal-regulated kinases
FEP: First-episode psychosis
FGAs: First generation antipsychotics
GABA: Gamma-amino butyric acid
GALP: Galanin-like peptide
GSK: Glycogen synthase kinase
HPA: Hypothalamic-pituitary-adrenal axis
HT: Hydroxytryptamine
IRS: Insulin receptor substrates
JAK: Janus activated kinase
*LEP*: Leptin gene
LepR: Leptin receptor
*LEPR*: Leptin receptor gene
MC: Melanocortin
MCH: Melanin-concentrating hormone
MDD: Major depressive disorder
NMDA: *N*-methyl-D-aspartate
NO: Nitric oxide
NPY: Neuropeptide Y
NT: Neurotensin
*OB*: Obese gene
*OBR*: Obese receptor gene
PFC: Prefrontal cortex
PI3K: Phosphatidylinositol 3-kinase
POMC: Pro-opiomelanocortin
PTP1b: Protein tyrosine phosphatase 1b
SGAs: Second generation antipsychotics
SOCS: Suppressor of cytokine signaling
STAT: Signal transducer and activator of transcription
TD: Tardive dyskinesia
